# Ultrasmall iron oxide nanoparticles induced ferroptosis via Beclin1/ATG5-dependent autophagy pathway

**DOI:** 10.1186/s40580-021-00260-z

**Published:** 2021-04-02

**Authors:** Jian Wen, Hanren Chen, Zhongyu Ren, Peng Zhang, Jianjiao Chen, Shulian Jiang

**Affiliations:** 1 Affiliated Hospital of Guilin Medical University, Guilin Medical University, Guilin Medical 26, University, 15 Lequn road, Guilin, 541000 People’s Republic of China; 2 Nanjing Second Hospital, 1 Zhongfu road, Nanjing, 210003 People’s Republic of China

**Keywords:** Autophagy, Ferroptosis, Ultrasmall iron oxide nanoparticle, Autolysosome

## Abstract

Iron-based nanoparticles, which could elicit ferroptosis, is becoming a promising new way to inhibit tumor cell growth. Notably, ultrasmall iron oxide nanoparticles (USIONPs) have been found to upregulate the autophagy process in glioblastoma (GBM) cells. Whether USIONPs could also elicit ferroptosis and the relationship between the USIONPs-induced autophagy and ferroptosis need to be explored. In the current study, our synthesized USIONPs with good water solubility could significantly upregulate the ferroptosis markers in GBM cells, and downregulate the expression of anti-ferroptosis genes. Interestingly,ferrostatin-1 could reverse USIONPs- induced ferroptosis, but inhibitors of apoptosis, pyroptosis, or necrosis could not. Meanwhile, autophagy inhibitor 3-methyladenine could also reverse the USIONPs-induced ferroptosis. In addition, shRNA silencing of upstream genes Beclin1/ATG5 of autophagy process could significantly reverse USIONPs-induced ferroptosis, whereas overexpression of Beclin1/ATG5 of autophagy process could remarkably promote USIONPs-induced ferroptosis. Furthermore, lysosome inhibitors could significantly reverse the USIONPs-induced ferroptosis. Collectively, these facts suggest that USIONPs-induced ferroptosis is regulated via Beclin1/ATG5-dependent autophagy pathway.

## Introduction

Glioblastoma (GBM), a malignant glioma, is the most common and devastating primary brain tumor in clinics, with an incidence rate as high as 60% in brain tumors. Due to the migration of GBM cells diffusely infiltrating into the surrounding normal brain tissue, GBM is easy to relapse after resection [1]. Despite comprehensive treatments such as surgery, radiotherapy and chemotherapy are currently used, the clinical outcomes of glioma patients are still very poor. Therefore, it is urgently to explore new ways to inhibit GBM cell growth and metastasis.

It is well known that most tumor cells have a high iron demand to mediate their rapid proliferation, and the concentration of iron ions in tumor cells is significantly higher than that of normal cells for the abnormal iron metabolism [2]. Notably, iron ions can produce free radicals including reactive oxygen species (ROS) through fenton reaction [3]. The sharp accumulation of ROS can lead to oxidative stress reaction, eventually causing serious oxidative damage or even death to the cells.

Ferroptosis, a new form of iron-dependent programmed cell death [4], is characteristic of higher levels of intracellular iron concentration, ROS, and lipid ROS. This death process can’t be suppressed by apoptosis, pyroptosis [5] and necrosis [6] inhibitors, but it can be inhibited by iron chelating agent and antioxidant [7, 8]. Therefore, induction of ferroptosis may be a potential novel anti-tumor method for GBM [9]. Recently, it has been demonstrated that iron-based nanoparticles could induce cell ferroptosis through lysosomal degradation pathway [9, 10]. Following the ingestion of iron-based nanoparticles by cells, excessive iron ions releasing from lysosome in an acidic environment stimulate the production of ROS through fenton reaction, consequently leading to cell ferroptosis.

Nowadays nanotechnology are widely used in all industrial domains, especially in the food and nanomedicine [11, 12]. There is no doubt that the risk of nanoparticles to human health and environment are gradually increasing due to their wide range of applications [13, 14]. Currently, clinical application of iron-based nano particles is the ultrasmall iron oxide nanoparticles (USIONPs, eg. Ferumoxytol,an iron agent) with hydrate particle size of less than 50 nm, which are not easily swallowed up by the Kupffer cells in the liver [15], thus leading to longer half-life in blood cycle than the larger sized IONPs. Of note, USIONPs can be enriched in tumor tissues via the high permeability and retention effect, through which patients could benefit from the tumor treatment [16–18]. Also nanoparticles could be modified to efficiently deliver in vivo across the blood–brain barrier and to target GBM [19, 20]. Up to now, however, no reports regarding the induction of ferroptosis by USIONPs in GBM cells have been documented. Thereby, it may be very important for potential application of clinical USIONPs in treatment of GBM patients.

Furthermore, the pathway related to USIONPs-induced ferroptosis needs to be clarified, which could greatly deepen the understanding of application of USIONPs in GBM treatment. Our previous studies reported that USIONPs could significantly up-regulate the expression of macroautophagy (hereafter referred to as autophagy)-related genes LC3-II and the LC3II/LC3I ratio in vitro, suggesting that USIONPs could active autophagy process [21]. Nevertheless, the relationship between autophagy pathway and USIONPs-induced ferroptosis remains to be clarified. Therefore, this research is to explore whether USIONPs could induce ferroptosis, and whether this induction process is regulated via autophagy pathway.

## Methods

### Preparation of USIONPs

Chemicals including acetone, ethanol, ferrous chloride, tris (acetylacetonato) iron(III), oleic, oleic acid, oil amine, and 1,2-distearoyl-sn-glycero-3-phosphoethanolamine-N- [amino (polyethylene glycol)-2000] (DSPE-PEG 2000) were purchased from Guoyao Chemical Incorporation (Shanghai, China).

**Fig. 1:**
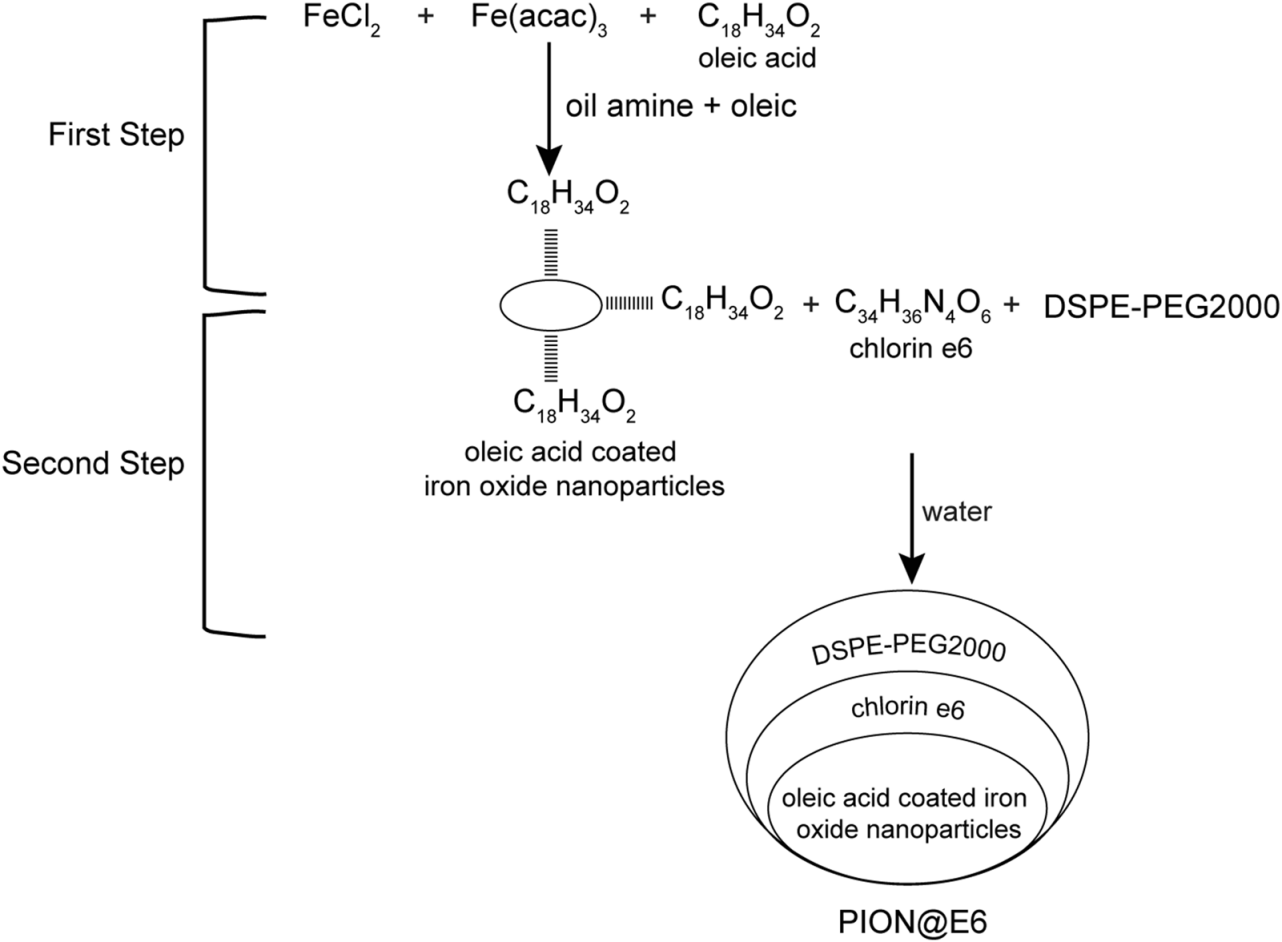
Two steps for synthesis of USIONPs(PION@E6). DSPE-PEG2000, 1,2-distearoyl-sn-glycero-3-phosphoethanolamine-N-[amino- (PEG)-2000]; E6, chlorin e6; PEG, polyethylene glycol; PION@E6, PEGylated iron oxide nanoparticles loaded with chlorin e6

As shown in Fig. 1, USIONPs(PION@E6) was synthesized according to our previously reported procedures [22]. Iron oxide nanoparticles (IONs) coated with oleic acid were prepared by means of high temperature pyrolysis. Briefly, 1.0 mmol FeCl_2_ solution, 6 mmol oleic acid and 6 mmol oil amine were added into 20 mL oleic by flushing the reaction medium with a nitrogen gas, then heated to 100–120℃ for 1 h. After that, 2.0 mmol Fe(acac)_3_ was added into the above mixture and heated to 180–220℃ for 30 min, then heated continuously for another 30 min to generate oleic acid coated IONs. After the reaction mixture cooled to room temperature, 75 mL anhydrous ethanol was added into the mixture to collect oleic acid coated IONs by magnetic separation. Following a washing with 35 mL acetone, the oleic acid coated IONs were collected by density-gradient centrifugation and then dissolved in 35 mL chloroform for the following preparation protocol.

To prepare USIONPs by the means of phase transfer, 50 mg DSPE-PEG 2000 and 10 mg chlorin e6(fluorescence tracer) were dissolved in 5 mL trichloromethane, and 10 mL the above collected oleic acid coated IONs was added into the mixture. After ultrasonic dispersion and addition of 5 mL deionized water, the mixture was rotarily evaporated to clear away the trichloromethane. After ultrasonic dispersion and cooling to room temperature, the supernatant aqueous phase solution containing USIONPs was collected via removing the aggregates by microfiltration and ultrafiltration.

### Characterization of USIONPs

The hydrate particle sizes and core particle size of PION@E6 were measured using a Particle Analysis Device (Brookhaven, USA) and Philips Transmission Electron Microscope (TEM; EM300, Philips, Netherlands), respectively. The zeta potential of PION@E6 was measured using a Zeta Potential Device (Nanjing Fuxin Analysis, China), and the stability of PION@E6 dissolved in deionized water was determined by Colloid stability analysis. Ultraviolet and visible spectrometry analysis was carried out using UV2700 UV–VIS Spectrophotometer(Shimazdu, Kyoto, Japan).

### Cell culture

Human GBM cell line U251 was obtained from the American Type Culture Collection (ATCC; Manassas, VA, USA). The cells were cultured in Dulbecco’s Modified Eagle Medium(DMEM) supplemented with 10% FBS, 100 U/mL penicillin and 100 µg/mL streptomycin in a humidified atmosphere of 5% CO_2_ incubator (Thermo Fisher Scientific, Waltham, MA, USA) at 37 °C. The cultivating media were refreshed every 3 d, and U251 cells in the logarithmic growth phase were used to conduct the experiments described as follows.

Rat glioma C6 cells were obtained from the American Type Culture Collection (ATCC; Manassas, VA) and cultured in DMEM supplemented with 10% FBS at 37 °C in a humidified incubator (Thermo Fisher Scientific, Waltham, MA, USA) under 5% CO_2_/95% air. The cells were changed with complete media every 3 d and routinely sub-cultured when the density of cells reached 80% confluence.

### Analysis of inhibition of cell proliferation

A cell-counting kit (CCK-8; Dojindo, Kumamoto, Japan) was used to measure cell proliferation according to the kit guideline. Optical densities (ODs) were measured at 450 nm using a microplate reader (Biorad, USA), and the cell inhibitory ratio was calculated according to the following formula:

$$\left[ {\left( {{1}{-}{\text{OD treated group}}} \right)/{\text{OD control group}}} \right]\, \times \,{1}00\%$$

### Measurement of cell proliferation using 5-ethynyl-2′-deoxyuridine (EdU)

Fluor488 Click-iT EdU imaging detection kit (KGA331-100, Kegen, Nanjing, China) was used to measure cell proliferation. Briefly, the cells were cultured in DMEM supplemented with 10% FBS in 96-well plates for 24 h. After washing cells with phosphate buffer saline (PBS) for two to three times,100 μL EdU (50 μmol/L) was added to the culture media for 2 h and then the cells were fixed with 4% paraformaldehyde for 30 min. After addition of 100 μL 1 × Apollo® staining buffer (excitation wavelength of 495 nm and emission wavelength of 520 nm) for 30 min, the cells were washed and added to 100 μL TritonX-100(0.5%). Thereafter, the cells were counterstained with 1 × Hoechst33342 buffer (excitation wavelength of 350 nm and emission wavelength of 461 nm) and imaged using a high content cell imaging system (200 ×) (MD, USA).

### Detection of lipid ROS

C11-BODIPY(Thermo, USA),as a fluorescent lipid peroxidation reporter molecule that shifts its fluorescence from red to green, was used to measure intracellular lipid ROS according to the instruction of manufacture. Briefly, after treatment with different concentrations of USIONPs for 48 h, the cells were then incubated with 100 μmol/L C11-BODIPY for 30 min at 37 °C. The samples were washed twice with PBS and then the fluorescence intensities were detected at an emission wavelength of 510 nm and an excitation wavelength of 488 nm using a Spectra Max M3 Fluorescence Microplate Reader (Molecular Devices, USA). Data were expressed as a percentage of the fluorescence intensity relative to vehicle controls.

### Measurement of intracellular ROS level

2′,7′-dichlorodihydrofluorescein diacetate(DCFH-DA) staining (Sigma, USA) was used to measure intracellular ROS. Briefly, after treatment with different concentrations of USIONPs for 48 h, the cells were incubated with 20 μmol/L DCFH-DA for 30 min at 37 °C. Thereafter, the cells were washed twice with PBS and the fluorescence intensities at an emission wavelength of 535 nm and an excitation wavelength of 485 nm were detected using a Spectra Max M3 Fluorescence Microplate Reader (Molecular Devices, USA). Data were expressed as a percentage of the fluorescence intensity relative to vehicle controls.

### Iron assay

Iron assay kit (MAK025, Sigma, USA) was used directly to detect both total and/or reduced iron concentrations in the samples after addition of acidic buffer. The released iron is reacted with a chromagen resulting in a colorimetric (593 nm) product, the intensity of which is proportional to the iron presented in the cells. After centrifugation at 16,000 × *g* for 10 min at 4 ℃, the supernatant was discharged. To measure total iron, 50 μL samples were supplemented with 5 μL iron reducer to reduce Fe^3+^ to Fe^2+^ and then adjusted to a final volume of 100 μL per well in a 96-well plate with assay buffer. Following a mixture on a horizontal shaker and incubation for 30 min at 25 ℃, 100 μL iron probe was respectively added to each well containing standard and test samples. Thereafter, the samples in the 96-well plate were mixed again and incubated for another 60 min at 25 ℃ in the dark. Finally, the absorbance was measured at 593 nm using a Spectra Max M3 Fluorescence Microplate Reader (Molecular Devices, USA).

### Western blotting assay

Cells were homogenized in lysis buffer, separated by 10% sodium dodecyl sulfate polyacrylamide gel electrophoresis and transferred to PVDF membranes (IPVH00010, EMD Millipore, Billerica, MA, USA). The membranes were blocked with 5% skimmed milk and then incubated with diluted primary antibodies including rabbit anti-FTH1(1:2000,abcam ab75973,UK), glutathione peroxidase 4(GPX4)(1:2000, ab125066, Aabcam, UK), Nrf2(1:1000, 16396–1-AP, Sanyin,Wuhan, China),Caspase-3(1:2000, ab184787, Abcam, UK),Bcl-2(1:2000, ab182858, Abcam, UK), Caspase-1(1:1000, ab207802,Aabcam, UK),NLRP3 (1:5000, ab210491, Aabcam, UK), Receptor interacting protein kinase (RIPK)3 (1:10,000, ab56164, Abcam UK), P62 (1:10,000; ab109012, Abcam, MA, USA), Beclin1(1:2000, ab207612, Abcam), LC3II (1:2000; ab192890, Abcam), LC3I (1:2000; ab192890, Abcam), and GAPDH (1:1000; ab181602, Abcam). The blots were washed three times with tris-buffered saline containing 0.1% (v/v) Tween-200 (TBST) and incubated with an appropriate goat peroxidase-conjugated secondary antibody (1:5000; KGAA35, Keygen Co., Nanjing, China) for 2 h. After washing three times with TBST, the blots were developed with the chemiluminescence method (ECL Luminata Crescendo, WBLUR0500, EMD Millipore).

### Silencing of Beclin 1 and ATG5 by the interference of shRNA

The lentivirus(LV)expressing Beclin 1 shRNA (Sense: 5ˊ-CCCGTGGAATGGAATGAGATT- 3ˊ; Antisense: 5ˊ-AATCTCATTCCATTCCACGGG-3ˊ) and ATG5 shRNA (Sense:5ˊ-CCTGAACAGAATCATCCTTAA-3ˊ; Antisense: 5ˊ-TTAAGGATGATTCTGTTCAGG-3ˊ) were generated and produced by Keygen Inc.Co. Ltd (Nanjing, China). Meanwhile, the empty vector was used as a control. After confirmation of the corrected insertions of shRNA cassettes by direct DNA sequencing, the shRNA-expressing LV was transfected into U251 cells together with the LV helper plasmids to generate respective LVs. Infectious LVs were harvested 48 h post-transfection, centrifuged to remove cell debris, and then filtered through 0.45 µm cellulose acetate filters. The transfection efficiency was determined by monitoring green fluorescent protein (GFP) expression. Moreover, the ability of the LV-shRNA-Beclin 1 and LV-shRNA-ATG5 vectors to knock down Beclin1 and ATG5 was investigated using quantitative polymerase chain reaction(qPCR).

### Overexpression of Beclin 1 and ATG5 by construction of lentivirus vectors

Human Beclin 1 and ATG5 genes were amplified by PCR and then cloned into the third- generation self-inactivating LV vector with cytomegalovirus promoter for driving constitutive expression of LV-Beclin 1 and LV-ATG5 vectors. The primers for Gene cloning were as follows, Beclin 1 (Sense: 5ˊ-TCCTCGAGACTAGTTaccatggaagggtctaagacgtc-3ˊ; Antisense:5ˊ-TAGTCCA TGGCGGCCgctttgttataaaattgtgagga-3ˊ), ATG5 (Sense:5ˊ-GATCTATTTCCGGTGaattcatgacagatg acaaagatgtg-3ˊ; Antisense: 5ˊ-TGGCGGCCGCTCTAGaatctgttggctgtgggatgatac-3ˊ). LVs expressing Beclin 1 and ATG5, and empty vector (as controls) were prepared by transient transfection in U251 cells. For all experiments, the cells were infected with LVs expressing wtα-syn at a multiplicity of infection of 40. After infection, the cells were cultured in a humidified, 5% CO_2_ atmosphere at 37 °C. All experiments were conducted in triplicate to ensure reproducibility.

### Verification by Real Time-PCR (RT-PCR)

Total RNA, including miRNAs, was extracted from U251 cells using the TRIzol reagent (Invitrogen, San Diego, CA, USA) according to the manufacturer's protocol. RNAs were reverse-transcribed using a cDNA First Strand cDNA Synthesis Kit(TaKaRa RR036B, Japan). For quantification, the One Step TB Green™ PrimeScript™ RT-PCR Kit II (SYBR Green) (TaKaRa RR086B, Japan) were utilized to perform qPCR following the manufacturer's instructions with a fluorescence qPCR thermal cycler (Step one plus RT-PCR system, ABI, USA). The expression of mRNA was defined from the threshold cycle (*C*_*t*_), and relative expression levels were calculated using the 2^−ΔΔCt^ method after normalization to the expression of GAPDH. RT-PCR primers are Beclin1 (Sense: 5ˊ-AATGGTGGCTTTCCTGGACT-3ˊ; Antisense: 5ˊ-TGATGGAATAGGAG CCGCCA-3ˊ), ATG5 ( Sense: 5ˊ-TGACGTTGGTAACTGACAAAG TG-3ˊ; Antisense:5ˊ-ATGCCATTTC AGTGGTGTGC-3ˊ) and GAPDH(Sense: 5ˊ-CAAATTCCA TGGCACCGTCA-3ˊ; Antisense:5ˊ-AGCATCGCCCCACTTGATTT-3ˊ).

### Statistical analysis

SPSS 13.0 (SPSS Inc., Chicago, USA) was used as the statistical analysis software. Statistical analyses were performed using two-way analysis of variance, and statistical values are presented as means ± standard error. *P* < 0.05 was considered significant.

## Results

### Characterization of our synthesized USIONPs

**Fig. 2:**
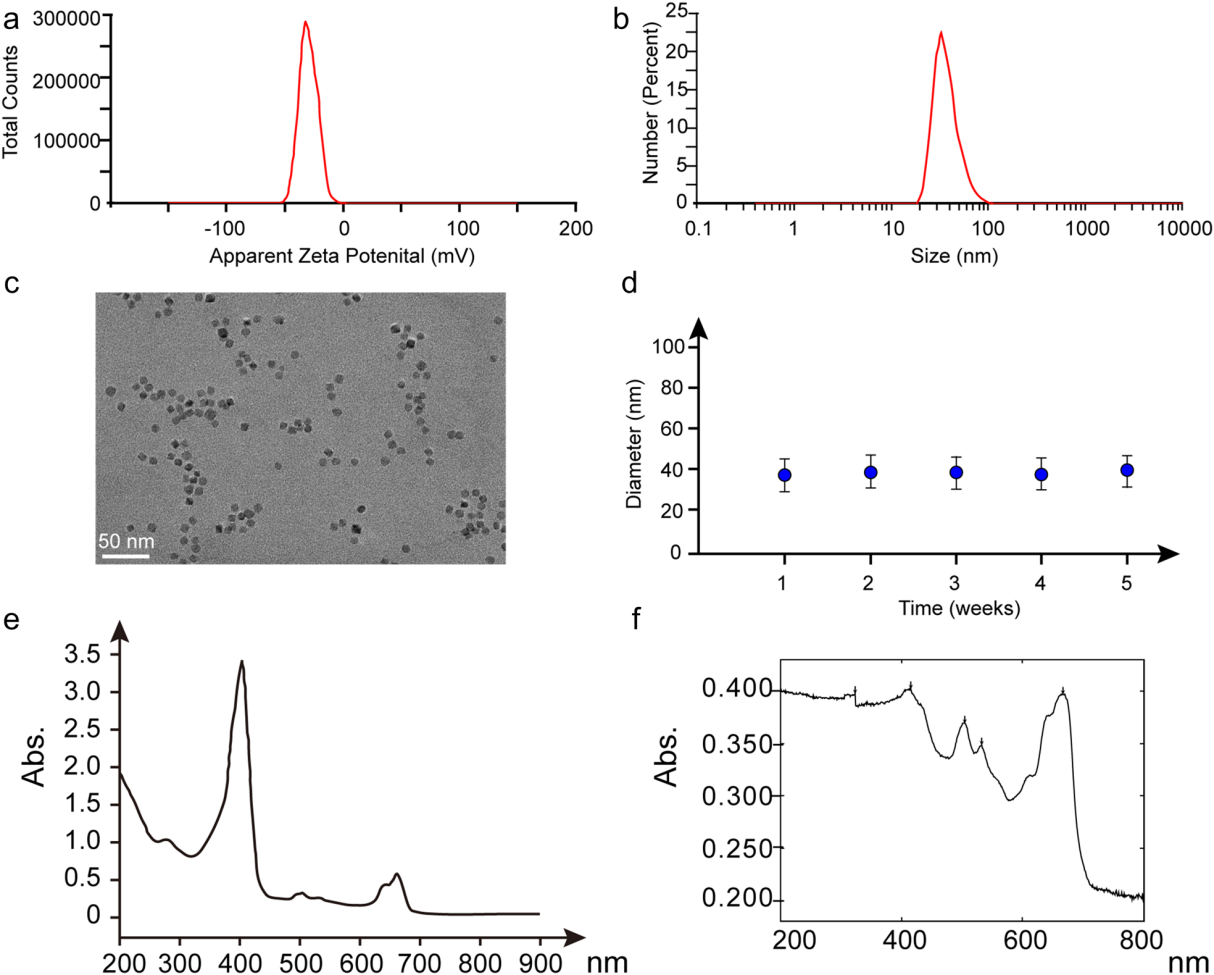
Characterization of IONPs. **a** Zeta potential of USIONPs using a Zeta Potential Device; **b** Hydrate particle size of USIONPs using a Particle Analysis Device; **c** Core particle size of USIONPs under TEM; **d** Colloid stability analysis of USIONPs; **e** Ultraviolet–visible absorption spectrometry analysis of chlorin E6; **f** Ultraviolet–visible absorption spectrometry analysis of USIONPs(PION@E6)

As shown in Fig. 2, zeta potential of our synthesized IONs was − 23.8 mV, the hydrate particle size was 37.86 ± 12.90 nm, and the core size was about 10 nm under TEM. Of note, colloid stability analysis showed no changes in the size of IONs after 5 W of still standing. Additionally, ultraviolet and visible absorption spectrometry analysis showed that fluorescent tracer chlorin e6 were successfully loaded onto the iron oxide nanoparticles.

### USIONPs-induced ferroptosis in GBM cells

**Fig. 3:**
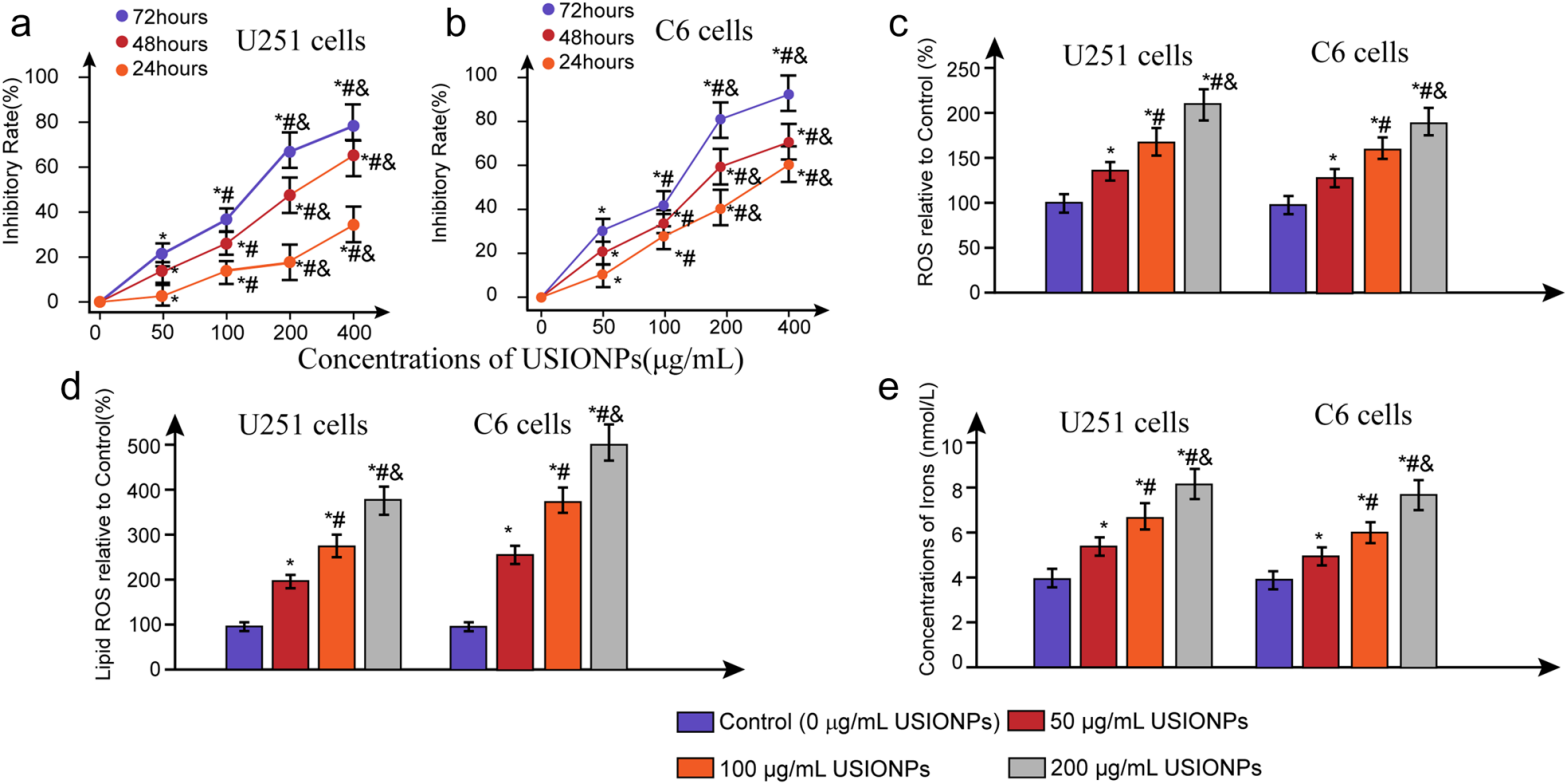
Effect of USIONPs on the cell inhibitory rates, intracellular ROS, concentrations of iron and ferroptosis in GBM cells. **a** Inhibitory rates of U251 cells treated with different concentrations of USIONPs for 24, 48, and 72 h; **b** Inhibitory rates of C6 cells treated with different concentrations of USIONPs for 24, 48, and 72 h; **c** Relative ROS levels in U251 cells and C6 cells treated with different concentrations of USIONPs for 48 h; **d** Relative lipid ROS levels in U251 cells and C6 cells treated with different concentrations of USIONPs for 48 h; **e** Iron concentrations in U251 cells and C6 cells treated with different concentrations of USIONPs for 48 h. *P < 0.05, statistically significant compared with control group. ^#^*P* < 0.05, statistically significant compared with 50 μg/mL USIONPs. ^&^*P* < 0.05, statistically significant compared with 100 μg/mL USIONPs

As shown in Fig. 3, USIONPs dose-dependently inhibited the proliferation of U251 cells and C6 cells compared with vehicle control (0 μg/mL USIONPs). However, there was no significantly difference in the inhibitory rate of U251 cells and C6 cells between 400 µg/mL USIONPs and 200 µg/mL USIONPs. Simultaneously, USIONPs could significantly induce the intracellular accumulation of irons accompanied with high levels of ROS and lipid ROS in both human GBM U251 cells and murine GBM C6 cells at 48 h., the effects of which were enhanced with an increasing concentration of USIONPs.

### USIONPs-induced ferroptosis reversed by ferrostatin-1(FER-1)

**Fig. 4:**
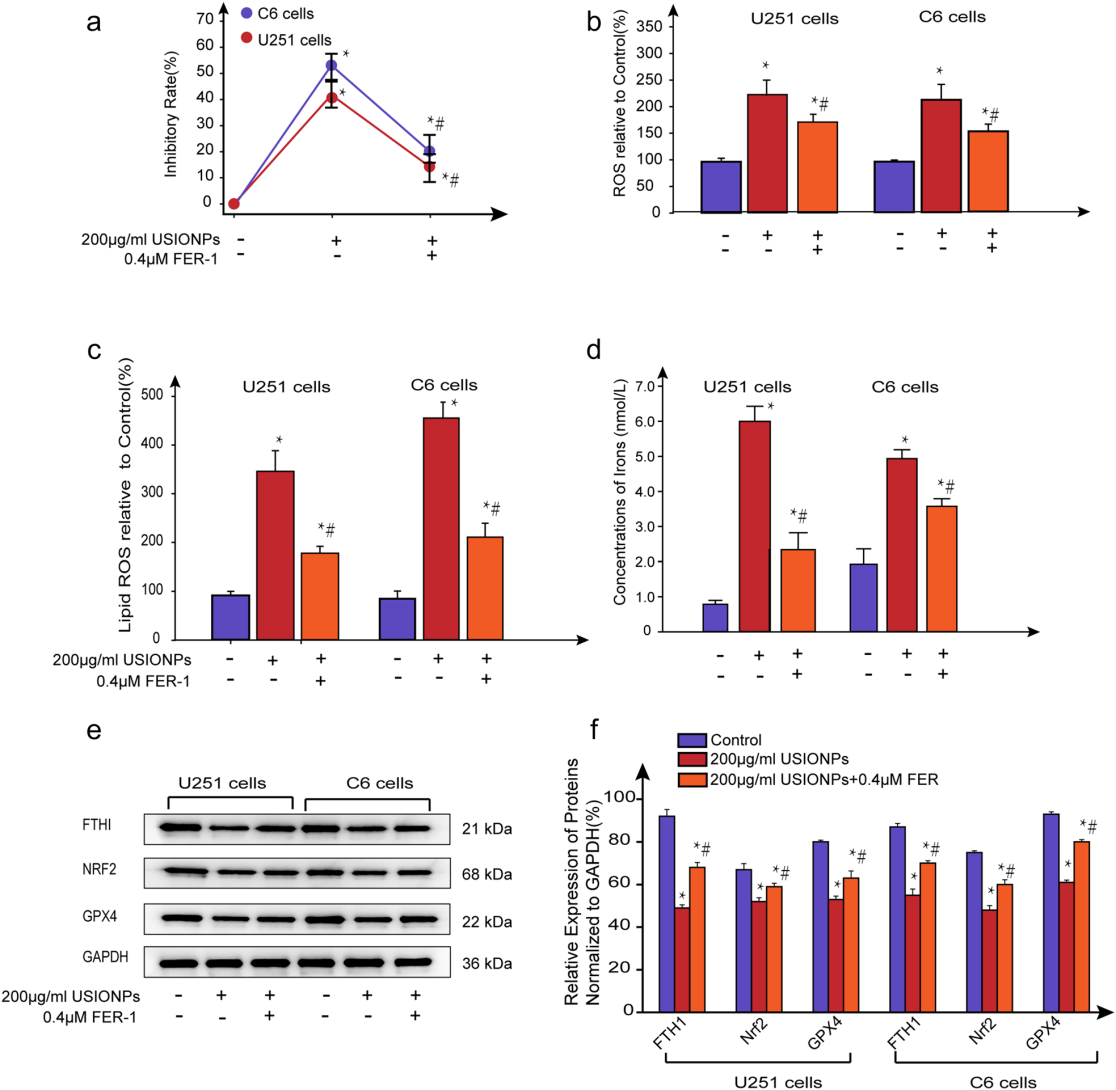
USIONPs-induced ferroptosis reversed by FER-1. **a** Inhibitory rates of U251 cells and C6 cells in exposure to USIONPs with or without 0.4 μmol/L FER-1 for 48 h; **b** Relative levels of ROS in the U251 cells and C6 cells in exposure to USIONPs with or without 0.4 μmol/L FER-1 for 48 h; **c** Relative levels of lipid ROS in the U251 cells and C6 cells in exposure to USIONPs with or without 0.4 μmol/L FER-1 for 48 h; **d** Concentrations of iron in the U251 cells and C6 cells in exposure to USIONPs with or without 0.4 μmol/L FER-1 for 48 h; **E** Representative images of ferroptosis related proteins in the U251 cells and C6 cells in exposure to USIONPs with or without 0.4 μmol/L FER-1 for 48 h; **f** Relative expression levels of proteins involved in the ferroptosis pathway in the U251 cells and C6 cells in exposure to USIONPs with or without 0.4 μmol/L FER-1 for 48 h. *P < 0.05, statistically significant compared with control group. ^#^*P* < 0.05, statistically significant compared with 200 μg/mL USIONPs

As shown in Fig. 4, the inhibition of U251 cells and C6 cells mediated by USIONPs could be significantly reversed after incubation with FER-1 for 48 h. In addition, the levels of intracellular ROS and lipid ROS, as well as irons of concentration upregulated by USIONPs were significantly inhibited after incubation with FER-1 for 48 h. Furthermore, the expression of anti-ferroptosis genes, including FTH1, NRF2, and GPX4 downregulated by USIONPs, were reversed after incubation with FER-1 for 48 h. All these suggest that FER-1 attenuates the USIONPs-induced ferroptosis in GBM cells.

### USIONPs-induced ferroptosis reversed by 3-methyladenine(3-MA) and not by inhibitors of apoptosis, necrosis and pyroptosis

**Fig.5:**
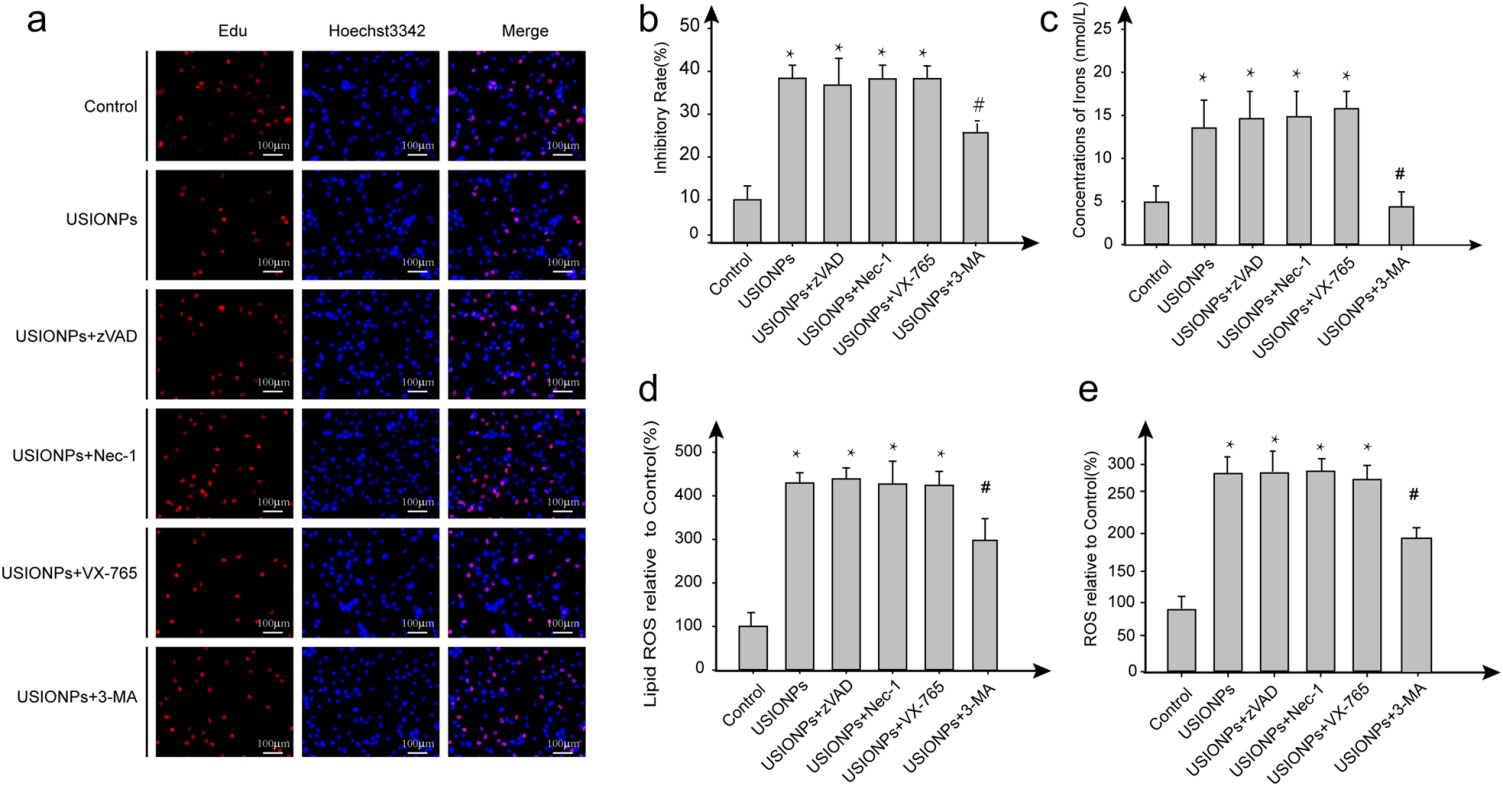
Effect on the USIONPs-induced ferroptosis after treatment with 200 μg/mL USIONPs, 20 μmol/L zVAD.fmk (apoptosis inhibitor), 20 μmol/L necrostatin-1 (necrosis inhibitor), 10 μmol/L VX765(pyroptosis inhibitor), and 10 μmol/L 3-MA (autophagy inhibitors) for 48 h. **a** Representative images of U251 cell proliferation by cell staining using Edu and Hoechst3342 (Magnification, 200 ×); **b** Inhibitory rates of U251 cells; **c** Concentrations of iron in the U251 cells; **d** Relative levels of lipid ROS in the U251 cells; (E)Relative levels of ROS in the U251 cells. *P < 0.05, statistically significant compared with control group

As shown in Fig. 5, the proliferation of U251 cells was significantly inhibited by USIONPs, and the effect of which couldn’t be reversed by the inhibitors of apoptosis, necrosis, and pyrotposis. Additionally, USIONPs-induced ferroptosis markers, including ROS, lipid ROS, and irons concentration, couldn’t be reversed by the inhibitors of apoptosis, necrosis, and pyrotposis. At the same time, inhibition of U251 cell proliferation mediated by USIONPs was reversed after incubation with autophagy inhibitor 3-MA for 48 h. Simultaneously, USIONPs-induced cell ferroptosis markers, including ROS, lipid ROS, and irons concentration, were reversed after incubation with 10 μmol/L 3-MA for 48 h.

### USIONPs-induced ferroptosis reversed by shRNA-mediated silencing of Beclin 1/ATG5

**Fig. 6:**
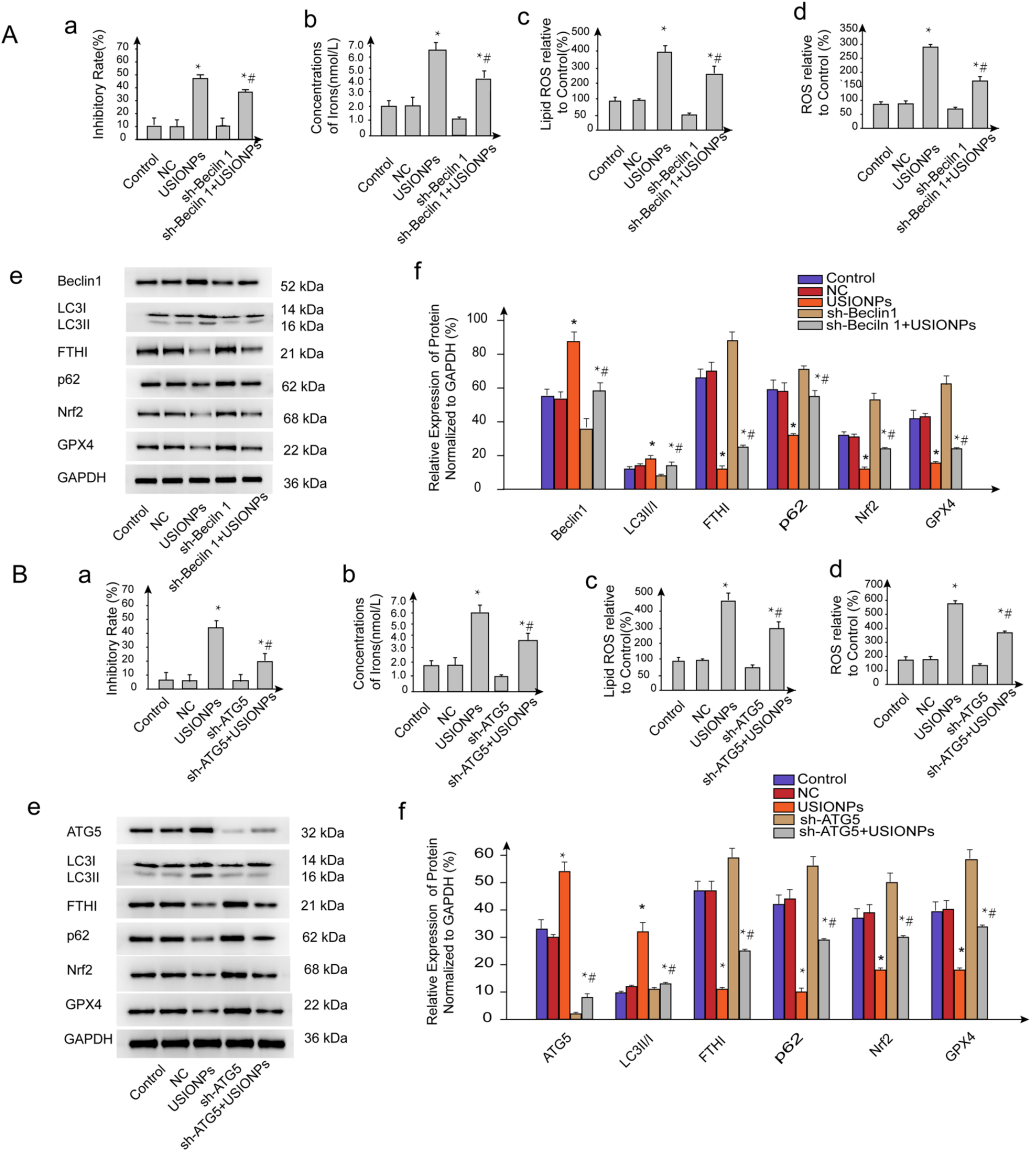
Reversal effect on the USIONPs-induced ferroptosis by the interference of Beclin 1/ATG5 for 48 h. **A**-**a** Inhibitory rates of U251 cells after silencing of Beclin1 gene; **A**-**b** Concentrations of iron in the U251 cells after silencing Beclin1 gene; **A**-**c** Relative levels of lipid ROS in the U251 cells after silencing Beclin1 gene; **A**-**d** Relative levels of ROS in the U251 cells after silencing Beclin1 gene; **A**-**e** Representative images of expression of autophagy and ferroptosis related proteins in the U251 cells after silencing Beclin1 gene; **A**-**f** Relative expression of autophagy and ferroptosis related proteins in the U251 cells after silencing Beclin1 gene; **B**-**a** Inhibitory rates of U251 cells after silencing ATG5 gene; **B**-**b** Concentrations of iron in the U251 cells after silencing ATG5 gene; **B**-**c** Relative levels of lipid ROS in the U251 cells after silencing ATG5 gene; **B**-**d** Relative levels of ROS in the U251 cells after silencing ATG5 gene; **B**-**e** Representative images of expression of autophagy and ferroptosis related proteins in the U251 cells after silencing ATG5 gene; **B**-**f** Relative expression of autophagy and ferroptosis related proteins in the U251 cells after silencing ATG5 gene. *P < 0.05, statistically significant compared with control group. ^#^*P* < 0.05, statistically significant compared with 200 μg/mL USIONPs

As shown in Fig. 6, inhibitory rate of U251 cells mediated by USIONPs was reversed by shRNA interference of Beclin 1/ATG5. Simultaneously, USIONPs-induced ferroptosis markers, including intracellular ROS, lipid ROS, and irons concentration, were reversed by the shRNA interference of Beclin1/ATG5.Additionally, Western blotting assay further demonstrated that shRNA interference of Beclin1/ATG5 significantly downregulated the expression of Beclin1/ATG5 and LC3II/LC3I ratio, and significantly upregulated the expression of p62,FTHI, Nrf2, and GPX4.

### Stimulation of USIONPs-induced ferroptosis by overexpression of Beclin 1/ATG5

**Fig.7:**
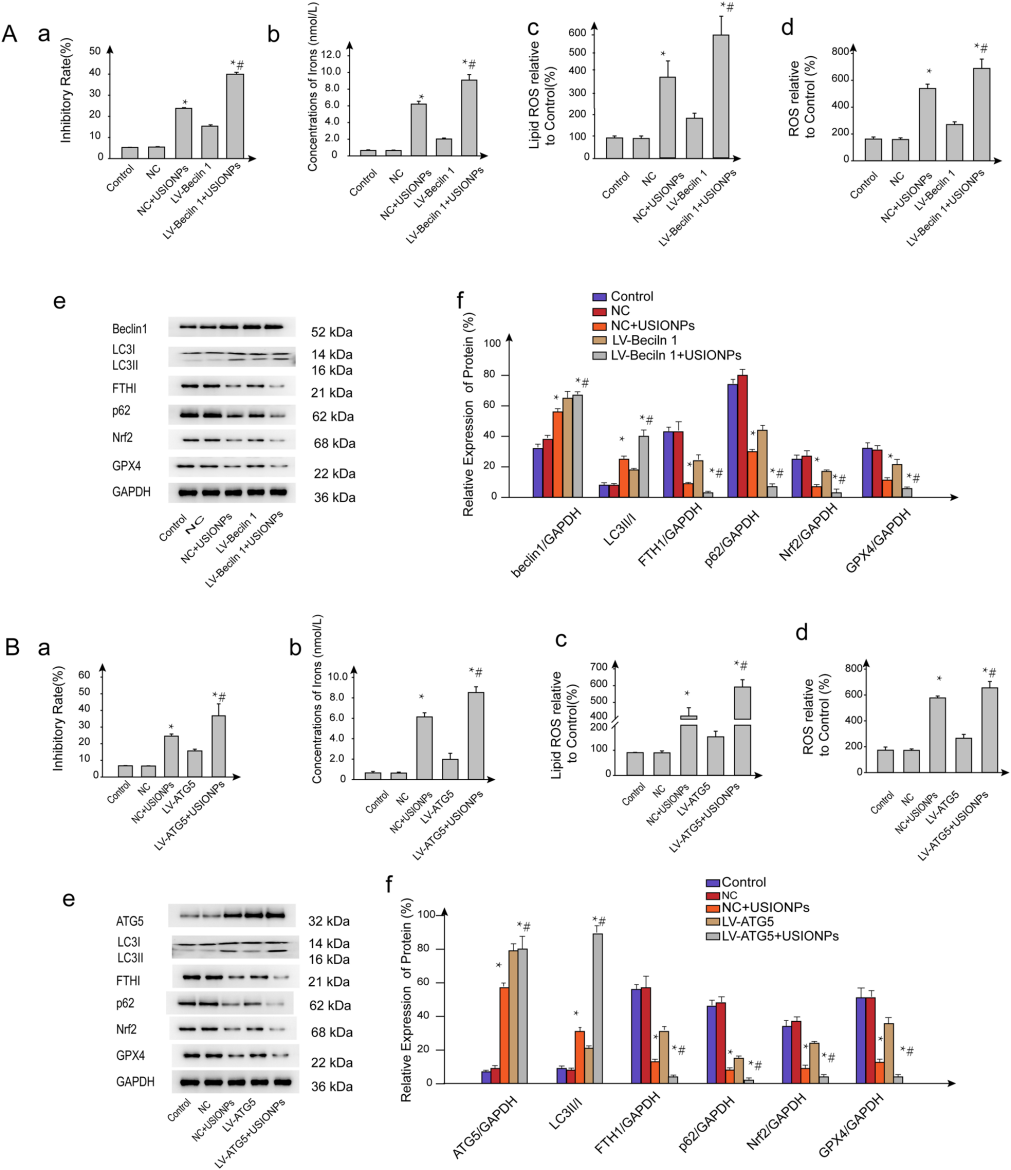
Stimulation of USIONPs-induced ferroptosis by the overexpression of Beclin 1/ATG5. **A**-**a** Inhibitory rates of U251 cells after overexpression of Beclin1 gene for 48 h; **A**-**b** Concentrations of iron in the U251 cells after overexpression of Beclin1 gene for 48 h; **A**-**c** Relative levels of lipid ROS in the U251 cells after overexpression of Beclin1 gene for 48 h; **A**-**d** Relative levels of ROS in the U251 cells after overexpression of Beclin1 gene for 48 h; **A**-**e** Representative images of expression of autophagy and ferroptosis related proteins in the U251 cells after overexpression of Beclin1 gene for 48 h; **A**-**f** Relative expression of autophagy and ferroptosis proteins in the U251 cells after overexpression of Beclin1 gene for 48 h; **B**-**a** Inhibitory rates of U251 cells after overexpression of ATG5 gene for 48 h; **B**-**b** Concentrations of iron in the U251 cells after overexpression of ATG5 gene for 48 h; **B**-**c** Relative levels of lipid ROS in the U251 cells after overexpression of ATG5 gene for 48 h; **B**-**d** Relative levels of ROS in the U251 cells after overexpression of ATG5 gene for 48 h; **B**-**e** Representative images of expression of autophagy and ferroptosis proteins in the U251 cells after overexpression of ATG5 gene for 48 h; **B**-**f** Relative expression of autophagy and ferroptosis related proteins in the U251 cells after overexpression of ATG5 gene for 48 h. *P < 0.05, statistically significant compared with control group. ^#^*P* < 0.05, statistically significant compared with 200 μg/mL USIONPs

As shown in Fig. 7, inhibitory rate of U251 cells mediated by USIONPs was further promoted by the overexpression of Beclin1/ATG5.Of note,USIONPs-induced ferroptosis markers, including intracellular ROS, lipid ROS, and irons concentration, were significantly upregulated by overexpression of Beclin1/ATG5. In addition, Western blotting assay further demonstrated that overexpression of Beclin1/ATG5 significantly upregulated the expression of Beclin1/ATG5 and LC3II/LC3I ratio, whereas significantly downregulated the expression of p62, FTHI, Nrf2, and GPX4. Furthermore, overexpression of Beclin1/ATG5 significantly promoted the expression of USIONPs-induced ferroptosis related proteins.

### USIONPs-induced ferroptosis influenced by lysosome inhibitors

**Fig. 8:**
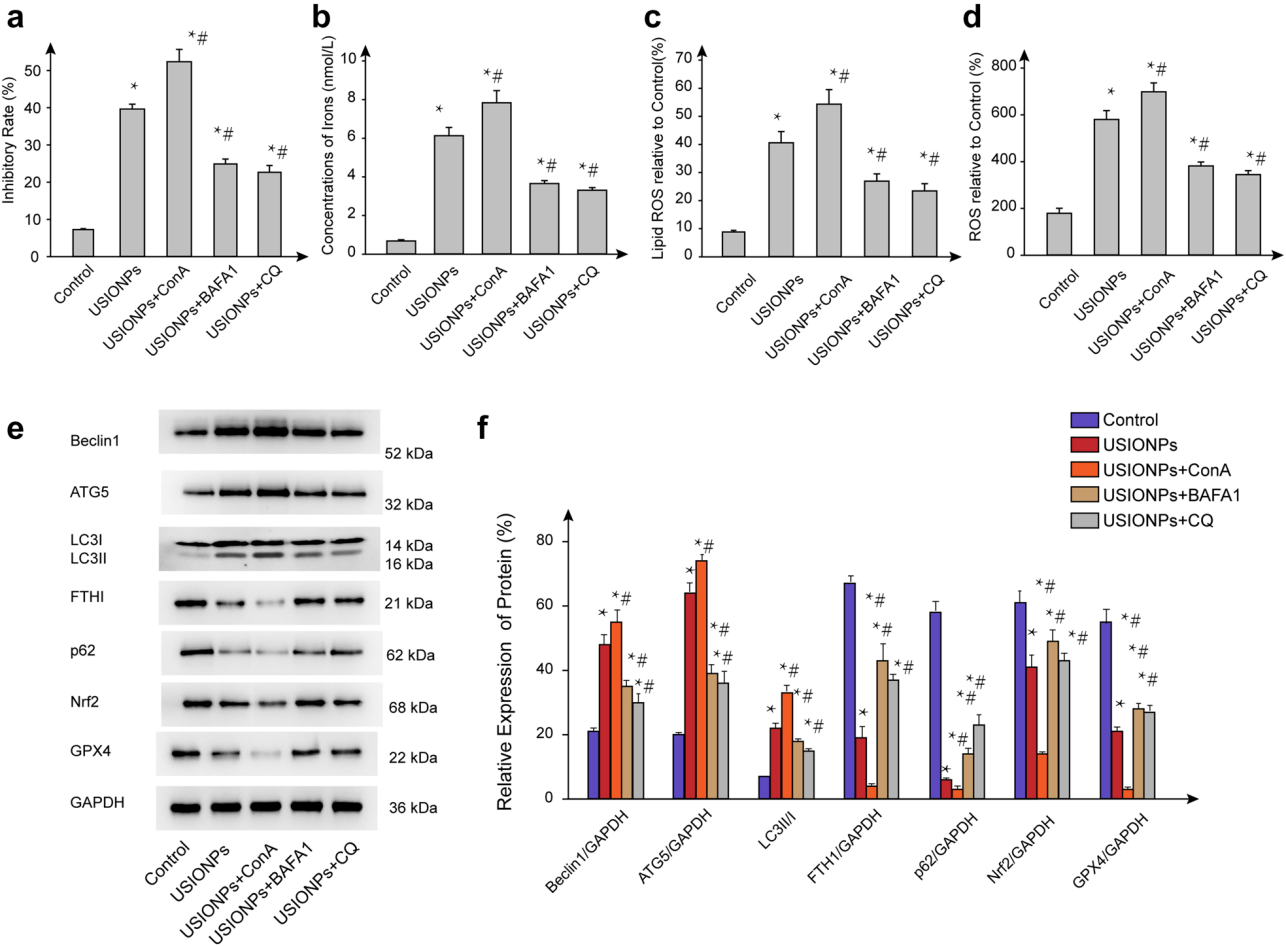
USIONPs-induced ferroptosis influenced by lysosome inhibitors for 48 h. **a** Inhibitory rates of U251 cells in exposure to USIONPs with or without lysosome inhibitors (ConA, BAFA1, and CQ); **b** Concentrations of iron in the U251 cells in exposure to USIONPs with or without lysosome inhibitors; **c** Relative levels of lipid ROS in the U251 cells in exposure to USIONPs with or without lysosome inhibitors; **d** Relative levels of ROS in the U251 cells in exposure to USIONPs with or without lysosome inhibitors; **e** Representative images of expression of autophagy and ferroptosis related proteins in the U251 cells in exposure to USIONPs with or without lysosome inhibitors; **f** Relative expression of autophagy and ferroptosis related proteins in the U251 cells in exposure to USIONPs with or without lysosome inhibitors.*P < 0.05, statistically significant compared with control group. ^#^*P* < 0.05, statistically significant compared with 200 μg/mL USIONPs

As shown in Fig. 8, inhibitory rate of U251 cells induced by USIONPs was reversed after incubation with lysosome inhibitors (BAFA1, CQ) for 48 h. Notably, USIONPs-induced ferroptosis markers, including intracellular ROS, lipid ROS, and irons concentration, were reversed after incubation with either BAFA1 or CQ for 48 h. Furthermore, Western blotting assay showed that both BAFA1 and CQ significantly suppressed the expression of autophagy related proteins (Beclin1, ATG5, and LC3II/LC3I ratio) upregulated by USIONPs, whereas promoted the expression of anti-ferroptosis related proteins (FTH1, NRF2, and GPX4) downregulated by USIONPs.

### Schematic illustration of underlying mechanism

**Fig. 9:**
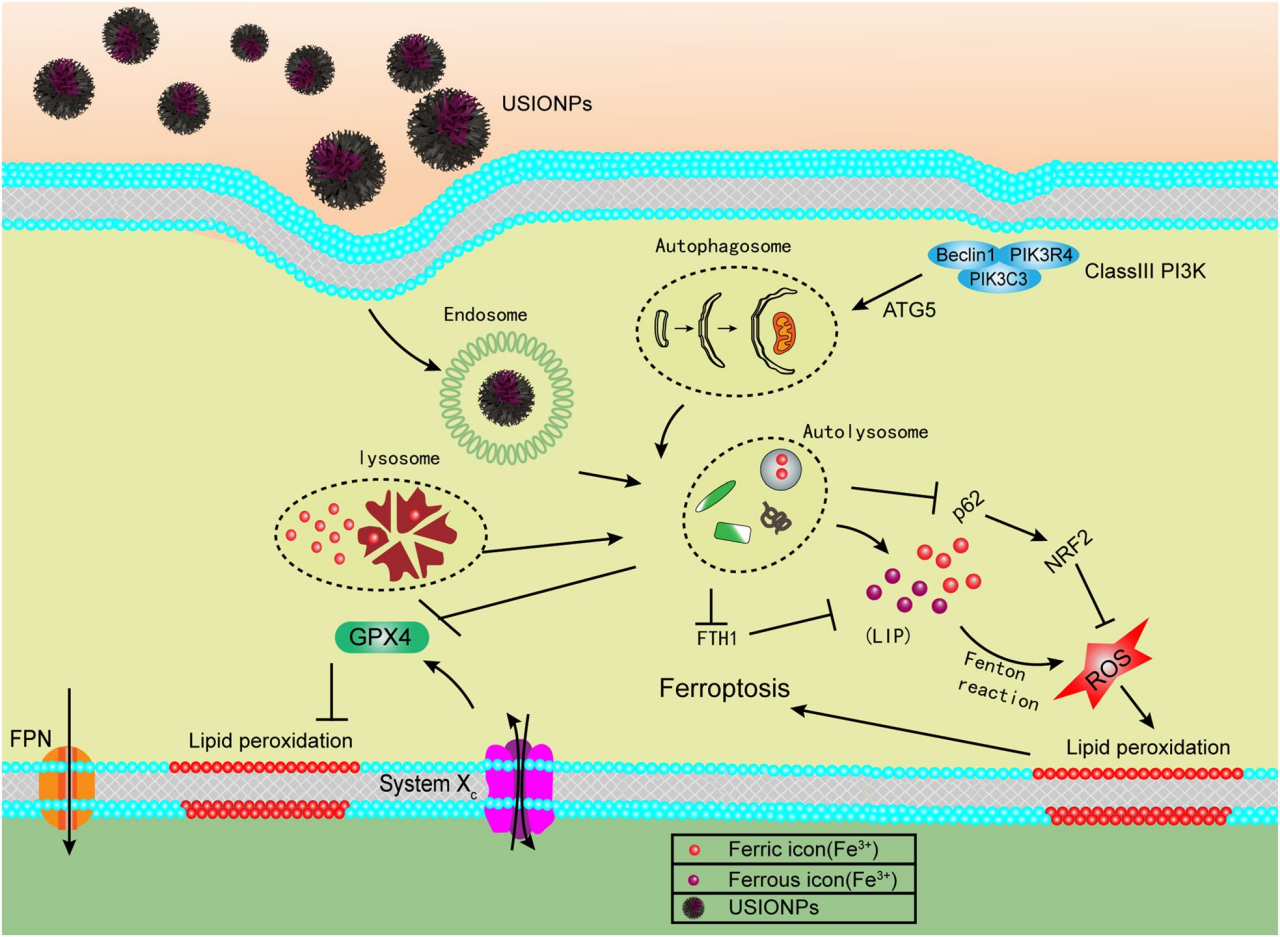
Schematic illustration of possible underlying mechanism responsible for the ultrasmall iron oxide nanoparticles-induced ferroptosis

As shown in Fig. 9, USIONPs may enter into cell via endocytosis, merge with atuophagosome, and further merge with lysome to form autolysosome, which release irons into labile iron pool, subsequently leading to accumulation of ROS and lipid oxidation, and eventually inducing the ferroptosis of GBM cells. Collectively, the molecular mechanism that USIONPs could upregulated autophagy genes and downregulated anti-oxidant genes may be responsible for the ultrasmall iron oxide nanoparticles-induced ferroptosis.

## Discussion

As shown in Fig. 2, PEGylation endowed our synthesized IONPs with long and stable good disperisity in aqueous phase. Notably, our synthesized IONPs owned a core size of 10 nm with a hydrate particle size of 37.86 ± 12.90 nm. All these facts suggest that the PEG-coated ultrasmall IONPs may be suitable for cell experiments owning to their average hydrate particle size of less than 50 nm in diameter and good disperisity.

As a new regulated way of programmed cell death, ferroptosis is different from apoptosis [6], pyroptosis [5], and necrosis [4], and it results from iron-dependent lipid peroxide accumulation. As we know, necrosis is mainly initiated by the tumor necrosis factor receptor family and the toll-like receptor family which is mainly recruited and phosphorylated by the mixed spectrum kinase domain-like protein mediated by the receptor RIPK1 and RIPK3, subsequently forms necrotic bodies and causes cell death [23]. Pyroptosis, which is mediated by cysteine aspartate-specific protease (Caspase 1), plays a pivotal role in the activation of host inflammatory response mainly by activating nucleotide-binding and oligomerization domain-like receptors, especially NLRP3 inflammosome [5]. As an emerging form of iron-dependent cell death, ferroptosis is mainly caused by the excessive accumulation of irons and lipid peroxides although the specific downstream effector proteins (e.g. pore-forming proteins) and the precise mechanisms remain unidentified. This death process is characterized by the increased iron content, ROS and lipid ROS in cytoplasm, smaller mitochondria, and higher mitochondrial membrane density. Intriguingly, ferroptosis could be suppressed by iron chelating agents and antioxidants, but not by inhibitors of apoptosis, pyroptosis, and necrosis [24]. Our results showed that our synthesized USIONPs significantly induced an increase in ferroptosis markers including irons concentration, intracellular ROS and lipid ROS. Of note, the inhibitors of apoptosis, pyroptosis, and necrosis showed no effect on the USIONPs-induced ferroptosis markers, including cell inhibition, irons concentration, intracellular ROS, and lipid ROS. Collectively, these facts suggest that ferroptosis is responsible for the regulation of death process induced by USIONPs but not apoptosis, pyroptosis, and necrosis.

FER-1, a synthetic antioxidant, is a potent and selective ferroptosis inhibitor that prevented accumulation of cytosolic and lipid ROS, and inhibited lipid peroxidation via a reductive mechanism to prevent damage to membrane lipids and thereby inhibited ferroptosis. Our experiments showed that after incubation with FER-1, the irons concentration was decreased. Also our experiments showed that FER-1 could significantly upregulate the FTH-1, which could restore iron. These facts suggest that FER-1 may decrease the irons concentration by upregulation of FTH-1.

Autophagy, an evolutionarily conserved self-degradative process, is initiated by the formation of omegasome from the endoplasmic reticulum. The omegasome then forms an isolated membrane that further expands and engulfs intracellular components, and subsequently fuses with the lysosome/vacuole, leading to the degradation of the engulfed cargos, eventually completing autophagy, through which the cells maintain homeostasis and genomic stability by scavenging oxygen free radicals and damaged organelles [25]. Mechanistically, Beclin1 is a key protein for autophagy initiation, which, together with PIK3C3 and PIK3R4, forms a protein complex Class III PI3K, and ultimately regulates the formation and maturation of autophagosomes [26–28]. Autophagy-related gene 5 is a gene product required for the formation of autophagosomes under the conditions of starvation or rapamycin blockage [29]. Concomitantly, a soluble cytosolic form of LC3 (LC3-I), when autophagy occurs, converts into a fat-soluble LC3-II, which is recruited to the autophagy body membrane for the formation of autophagosomes. Using multispectral imaging flow cytometry, the expression of LC3-II has been demonstrated as autophagy activity markers [30]. We reported in our previous research that USIONPs can up-regulate the expression of Beclin1 and the ratio of LC3II/LC3I, thereby it can be inferred that USIONPs can enhance autophagy to inhibit cell invasion [21]. In this research, we also demonstrated that USIONPs could upregulate the expression of autophagy related proteins, the effect of which was suppressed by autophagy inhibitor 3-MA. Simultaneously, 3-MA significantly inhibited the USIONPs-induced ferroptosis. Our further research demonstrated that inhibition of autophagy by shRNA knock-down of Beclin1/ATG5 could significantly suppress the USIONPs-induced ferroptosis, whereas overexpression of ATG5/Beclin1 could significantly enhance the USIONPs-induced ferroptosis. These facts suggest that autophagy may play an important role in USIONPs-induced ferroptosis. Notably, ferroptosis is strongly dependent of USIONPs uptake because the USIONPs can supply intracellular iron ions. Thus, whether autophagy inhibitors used in this study affects the cellular uptake of NPs needs further evaluation in our future studies.

Although the molecular details are still not completely understood, FTH1, GPX4, and NRF2 are involved in the autophagy-dependent ferroptosis [31]. A previous study verified that inhibition of FTH1 gave rise to iron overload and increased the rate of cell ferroptosis, suggesting that FTH1 is probably involved in the ferroptosis [31]. GPX4, which is known to have an inhibitory effect on 12-lipoxygenase activity, converts the reduced intracellular glutathione into oxidized glutathione, promotes H_2_O_2_ decomposition, thus protecting cell membranes from damage by oxides [32]. More importantly, the transcription factor Nrf2 and Kelch-like ECH-associated protein1 (Keap1) signaling pathways play an important role in preventing cells from oxidative stresses. The two cellular pathways were certificated to intersect through the direct interaction between p62 (an autophagy adaptor protein) and Keap1 (the Nrf2 substrate adaptor for the Cul3/E3 ubiquitin ligase) [33]. For instance, downregulation of p62 increased the level of Keap1, which decreased Nrf2 activation, downregulated gene expression of cytoprotective antioxidant, and caused ferroptosis. This suggests that USIONPs may induce ferroptosis by strengthening the autophagy process, during which the p62 was degradated, subsequently leading to downregulation of Nrf2 and lower level of cytoprotecitve antioxidant[31]. It is further demonstrated that the anti-ferroptosis related proteins, including FTH1, GPX4, and NRF2, were significantly suppressed after incubation with USIONPs for 48 h, the effect of which could be reversed by a potent and selective ferroptosis inhibitor FER-1. These facts suggest that these proteins are possibly cleared away by USIONPs-induced autophagy, therefore inhibition of autophagy could reverse this effect.

So far, the two commonly used inhibitors of autolysosomal function are BAFA1 and CQ. BAFA1 is a known inhibitor of the late phase of autophagy that prevents maturation of autophagic vacuoles by inhibiting late stage fusion between autophagosomes and lysosomes. Whilst CQ mainly blocks late-stage autophagy by impairing autophagosome–lysosome fusion rather than by affecting the acidity and/or degradative activity of this organelle. In the current study, both BAFA1 and CQ could significantly inhibit the USIONPs-induced ferroptosis. It was previously reported that the degradation of iron oxide nanoparticles by lysosomes after endocytosis can lead to the release and accumulation of iron ions and the production of ROS through fenton reaction with peroxides such as H_2_O_2_ [34]. On the other hand, ConA is an antibiotic and well-noted specific inhibitor of V-ATPase activity to block the acidification of lysosomes [35]. Our findings demonstrated that ConA showed quite opposite effects in comparison with other lysosome inhibitors BAFA1 and CQ. These facts suggest that fusion between autophagosomes and lysosomes may play a much more important role in USIONPs-induced ferroptosis than the acidification of lysosomes, which needs further clarification in our future research.

Simultaneously, our experiments showed that inhibitors of apoptosis, necrosis, and pyroptosis cannot reverse USIONPs-induced ferroptosis. However, ConA, a specific inhibitor of vacuolar type H + -ATPase activity (V-ATPase),which is capable of inducing apoptosis[36], promoted USIONPs-induced cell inhibitory rates unlike BAFA1 and CQ. These suggested that inhibitor of apoptosis couldn't reverse the USIONPs-induced ferroptosis, while apoptosis inducer may help USIONPs-induced ferroptosis. However, the underlying mechanism needs further elucidation in our future researches.

Altogether, USIONPs possibly induce ferroptosis via upregulation of autophagy process, which is responsible for the degradation of USIONPs and release of iron ions, subsequently leading to the accumulation of ROS and lipid peroxidation, and eventually inducing the ferroptosis of GBM cells.

## Conclusion

These facts suggest that USIONPs-induced ferroptosis is possibly regulated via Beclin1/ATG5 dependent autophagy process.

## Data Availability

The datasets used and/or analyzed during the current study are available from the corresponding author on reasonable request.

## Abbreviations

ATG5: Autophagy-related gene 5
BAFA1: Bafilomycin A1
CCK-8: Cell-counting kit
conA: Concanamycin A
CQ: Chloroquine
DCFH-DA: 2′,7′-Dichlorodihydrofluorescein diacetate
DMEM: Dulbecco’s modified eagle medium
FER-1: Ferrostatin-1
FPN: Ferroportin
GBM: Glioblastoma
IONs: Iron oxide nanoparticles
LIP: Labile iron pool
3-MA: 3-Methyladenine
NC: Negative control
OD: Optical density
Abs: Absorptions
PBS: Phosphate saline buffer
PCR: Polymerase chain reaction
RIPK: Receptor interacting protein kinase
ROS: Reactive oxygen species
TEM: Transmission electron microscope
USIONPs: Ultrasmall iron oxide nanoparticles
